# Group rhythmic synchrony and attention in children

**DOI:** 10.3389/fpsyg.2013.00564

**Published:** 2013-09-02

**Authors:** Alexander K. Khalil, Victor Minces, Grainne McLoughlin, Andrea Chiba

**Affiliations:** ^1^Department of Cognitive Science, University of CaliforniaSan Diego, CA, USA; ^2^Institute of Psychiatry, King's CollegeLondon, UK

**Keywords:** attention, synchrony, music cognition, temporal processing, learning

## Abstract

Synchrony, or the coordinated processing of time, is an often-overlooked yet critical context for human interaction. This study tests the relationship between the ability to synchronize rhythmically in a group setting with the ability to attend in 102 elementary schoolchildren. Impairments in temporal processing have frequently been shown to exist in clinical populations with learning disorders, particularly those with Attention Deficit Hyperactivity Disorder (ADHD). Based on this evidence, we hypothesized that the ability to synchronize rhythmically in a group setting—an instance of the type of temporal processing necessary for successful interaction and learning—would be correlated with the ability to attend across the continuum of the population. A music class is an ideal setting for the study of interpersonal timing. In order to measure synchrony in this context, we constructed instruments that allowed the recording and measurement of individual rhythmic performance. The SWAN teacher questionnaire was used as a measurement of attentional behavior. We find that the ability to synchronize with others in a group music class can predict a child's attentional behavior.

## Introduction

The ability to attend is among the most important skills that children must develop and refine. In an interpersonal context, the ability to attend may be related to the ability to synchronize, which we define as the generation of behaviors in coordination with an external stimulus at specific points in time. Co-attention with a parent emerges in infancy (Deák et al., 2000; de Barbaro et al., 2000) and naturally requires an infant to synchronize, directing gaze, and orientation nearly in unison with the parent. Later, in more complex forms of interaction such as conversation, the ability to synchronize facilitates smooth transfer of information (Bernieri and Rosenthal, 1991).

Studies of attention deficit hyperactivity disorder (ADHD) suggest a link between attention and temporal processing, as reviewed by Toplak et al. (2006). Children with ADHD who by definition display inattentive behavior have been shown to perform more poorly than controls on a variety of temporal processing tasks, such as duration discrimination, duration reproduction, anticipation tasks, and finger tapping tasks (Gilden and Marusich, 2009). Impaired temporal processing has been posited as a cognitive marker of ADHD (Castellanos and Tannock, 2002). Similarly, variability in response times, or reaction time variability (RTV), has been identified as increased in ADHD across a variety of tasks and contexts (Gilden and Marusich, 2009; Klein et al., 2006).

A useful measure of temporal processing can be found in rhythmic components of the practice of music. Rather than referring specifically to “beats,” rhythm in music refers to any form of temporal pattern, regardless of the presence of “beats.” Playing music requires one to maintain synchrony across multiple nested timescales, from tens of milliseconds to minutes. In particular, theoretical work suggests that synchronizing with an isochronous beat—a basic musical skill—requires the perception and estimation of time intervals in the order of 10 ms to 1000 ms. (Repp, 2005; Jacoby and Repp, 2012). The familiar context of a group music class may be an efficient and ecologically-valid method of measuring children's ability at such tasks.

The aim of this study was to examine the relationship between temporal processing, as measured by the ability of a child to synchronize to a driving beat, in the context of a music class, and attentional behavior, measured by teacher ratings. While the link between temporal processing and attentional behavior has been confirmed in samples diagnosed with ADHD, we do not yet know how temporal processing relates to attention in the general population. Based on the previous literature, we hypothesize that the ability of children to synchronize is correlated with their attentional behavior.

In order to measure the ability to synchronize, we use specially-wired instruments that allow detection of each mallet strike of each player. In order to measure attentional behavior, we use teacher ratings of the Strengths and Weaknesses of ADHD and Normal Behaviors (SWAN) questionnaire (Swanson et al., 2006). This DSM-IV based questionnaire was developed to assess ADHD-like symptoms across the continuum of children's behavior (rather than at the extreme, maladaptive end). We also included cognitive-performance measures (mean reaction time, RTV and errors) from a task that is often used in attentional and ADHD research, the Eriksen Flanker Task (EFT; Albrecht et al., 2008).

## Materials and methods

### Overview

Participants were scored along three different dimensions. Synchrony, pertaining to measurement of musical timing, the SWAN teacher rating questionnaire, which includes a behavioral measure of attention (Swanson et al., 2006), and the Eriksen flanker task, a computerized psychometric task often used to measure attentional control (Eriksen, 1995).

Our assessment of participants' rhythmic synchrony was conducted in a context that reflected a typical music class. Groups of roughly 12 participants playing five-keyed metallophones were seated in a semi circle facing a leader. The leader played a beat on a non-pitched percussion instrument. The synchrony of each participant was assessed [defined] against this beat.

A musical beat can be described by the movement of a point around the perimeter of a circle. The location of this point with respect to the center of the circle defines an angle. When playing an isochronous pulse, the exact time of the leader's onsets (mallet strikes) corresponds to an angle of 0, the quarter beat to an angle of π/2, the offbeat to π, and so forth. We calculated this time-dependent angle (or phase) based on the leader's onsets. We then calculated each participant's onsets and analyzed the distribution of the angles of their individual mallet strikes. The more narrow the distribution of angles, the more regular in relation to the leader a participant's playing. Such a player would be ranked more highly than one with a wider distribution of angles.

We then calculated the correlation of within-group synchrony rankings with attention behavior, as measured by the SWAN questionnaire and the Eriksen flanker task. In order to do this we calculated the correlation coefficients between the different measures. We corrected for performance differences across grades and genders by using them as covariates.

### Participants

We tested 102 students from grades 2–6 (Table 1) at the Museum School, a charter school in San Diego, CA. The ethnic composition of the participants was as follows: White 43%, Hispanic 31%, African American 13%, Asian/Pacific Islander 13%. Fifty-six percent were female. The number of students in each grade can be seen on Table 1. All of the participants had previous experience with musical training, as it was one of their curriculum classes (30 min-classes, once a week) and Dr. Khalil was their teacher. Here, it is important to note that Khalil is an ethnomusicologist with 25 years experience studying and/or teaching traditional Balinese gamelan music. Thus, despite his cross-training in behavioral and computational neuroscience (expertise held by the other members of the team), he approaches the classroom as a seasoned instructor.

**Table 1:** **Descriptive statistics**.

**Grade**	**Participants**	**VS**	**SWAN I**	**SWAN C**	**CONG**	**INC**	**RT CONG**	**RT INC**	**RTV CONG**	**RTV INC**	**RTD**
2	21	0.869 (0.13)	−4.52 (11)	−7.1 (22)	0.808 (0.18)	0.634 (0.18)	485 (78)	568 (1e + 02)	152 (59)	166 (78)	82.7 (40)
3	16	0.894 (0.054)	3.31 (7.9)	3.92 (15)	0.869 (0.11)	0.607 (0.21)	455 (90)	527 (91)	117 (53)	124 (38)	71.5 (17)
4	24	0.854 (0.095)	−2.48 (13)	−10 (26)	0.784 (0.2)	0.575 (0.23)	411 (69)	488 (73)	144 (63)	157 (92)	76.9 (32)
5	23	0.915 (0.041)	−3.62 (12)	−16.8 (23)	0.839 (0.19)	0.601 (0.24)	387 (79)	459 (90)	96.7 (31)	106 (55)	71.9 (23)
6	18	0.944 (0.02)	−0.667 (7.1)	−3.5 (13)	0.856 (0.2)	0.568 (0.23)	369 (60)	446 (54)	96.6 (52)	98.4 (54)	77.2 (25)

Average values of the different variables by grade, standard deviations in parenthesis. SWAN I and SWAN C are the mean scores in the mainly inattentive and mainly hyperactive SWAN questionnaires respectively. CONG and INC stand for proportion correct in the congruent and incongruent conditions respectively. RT is reaction time (in ms), RTV is within participant variability in reaction time, measured as standard deviation of reaction times. RTD is the difference in reaction time in the incongruent vs. congruent conditions.

### Rhythmic synchrony

**Figure 1:**
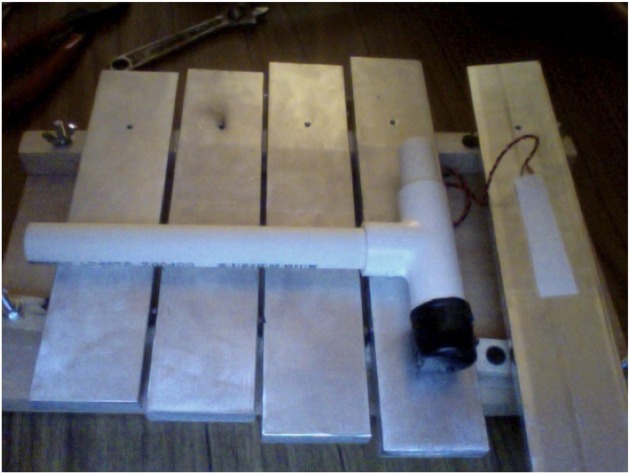
**Example of musical instrument constructed for this study.** These pitched-percussion instruments have a piezoelectric film element affixed to each key. This allows isolated recording of each instrument.

Gamelan is a musical art form that strongly emphasizes rhythmic synchrony among the ensemble members. A gamelan ensemble is made up of pitched percussion instruments with bronze keys. The instruments used for testing rhythmic synchrony were modeled after gamelan instruments, see Figure 1.

The gamelan-like instruments we designed and constructed feature piezoelectric film elements applied to each key. The importance of this innovation is that it allows each mallet strike on each key, for each player, to be recorded individually and in isolation in a temporally precise manner (details below). The instrument played by the instructor has a distinctively different timbre, or harmonic profile, than the ones used by participants.

Participants within each grade (2–6) were divided into two groups of equal size; if the number of participants within a particular grade was odd, one of the groups would have one more member. Participants were recorded as they attempted to synchronize with an isochronous beat played by the instructor over 1 min. Because our interest was in recording participants while engaged in the task, rather than recording them over a long period—during which time many participants could become disengaged—we repeated this process four times, interleaving these episodes with other musical activities across a 30-min recording session.

We calculated the synchrony between each player and the leader according to the following procedure. We first calculated each participant's onsets. In order to find the onsets of the leader we ran a complex filter on the audio signal at 200 Hz (50 Hz bandwidth) and took its absolute value. This process revealed clear peaks coinciding with the onsets. A threshold was chosen by hand through visual inspection based on the individual records. The first time a peak crossed the threshold was considered the time of the onset. If two peaks were observed over a period of 200 ms. we considered only the first one. We adopted this criterion in order to avoid counting “double-hits” caused by mallet bounce. A similar procedure was used to find the participants' onsets, except that 500 Hz (50 Hz bandwidth) was used as the filtering frequency due to the higher fundamental and harmonic profile of the participants' instruments.

We next calculated the phase of the leader. In order to do this we ran a complex filter on the leader's onset data centered on 1 Hz (bandwidth.75 Hz) and took the angle of the resulting signal. This produced a time-dependent phase signal that was 0 on the beat and π on the off-beat. A set of phases for each participant was collected by evaluating the phase signal obtained from the leader at each participant's onset times, or mallet strikes.

In order to measure the acuity of each participant's synchrony, we performed a vector strength (VS) analysis. This measure was introduced by Goldberg and Brown (1968) and has been extensively employed to analyze synchrony of neural activity.

VS is defined as follows:

$$VS=\frac{1}{N}\left|\sum_{j=1}^{N}e^{i^{*}\varphi j}\right|$$

Where ***N*** is the total number of onsets of one participant, j is the onset number, φ is the corresponding phase, and ***i*** is the imaginary unit. Thus, defined, VS is 1 if the participant always plays with the same phase with respect to the driving beat and 0 if she or he plays randomly. For each participant, we calculated the average *VS* across all four synchronization episodes.

VS more accurately reflects the ability to synchronize in a class setting than others that have been used for similar work, such as inter-tap variability. This is because it remains unaffected if a player misses a few beats or if the lead player changes tempo.

### Eriksen flanker task

The Eriksen Flanker Task (EFT; Eriksen, 1995) was administered to each participant. Each participant was asked to press a key with their left or right index finger when a central arrow (target) appeared in the middle of the screen pointing in the corresponding direction. 100 ms before the target, two flanking distractor arrows appeared that pointed either in the same (congruent condition) or opposite (incongruent condition) direction as the target arrow. We computed the percentage of correct responses as the number of correct responses over the total number of presentations for each condition (congruent and incongruent). For the calculation of reaction time measures we only considered correct responses. We calculated the mean reaction time for each condition; the RTV, measured as standard deviation; and the reaction time difference across conditions (incongruent—congruent). See below for a list of all variables.

### Swan questionnaire

The Strengths and Weaknesses of ADHD Symptoms and Normal Behavior (SWAN) Rating Scale (Swanson et al., 2006) is a questionnaire of the Likert-type based on the Diagnostic and Statistical Manual of Mental Disorders (4th ed; DSM-IV, APA, 1994). The questionnaire assesses children's behavior along the dimensions of inattention and hyperactivity-impulsivity. It was designed to be sensitive at both the negative and adaptive ends of the two symptom dimensions of ADHD. It was shown to have internal consistency and test-retest reliability (Arnett et al., 2012) as well as external validity (Arnett et al., 2012) as compared with the Disruptive Behavior Rating Scale (DBRS; Barkley and Murphy, 2006).

The SWAN is rated on a balanced 7-point Likert-type scale, with anchors far above, above, slightly above, average, slightly below, below, and far below. For measuring behavioral attention/inattention, SWAN includes items such as: “Compared to other children”… the child … “Sustains attention on tasks or play activities.” It is composed of eighteen questions. Nine of them assess attentive behavior (SWAN I) and the other nine address hyperactive-impulsive behavior (SWAN H). Therefore, SWAN I is a behavioral measure of attention. The total questionnaire is referred to as SWAN C, where C refers to inattentive and hyperactive combined and is considered a measure of ADHD-like behavior. This questionnaire was filled out for each participant by the homeroom teacher.

For analyzing the SWAN data, we numbered the anchors from −3 to 3 and calculated the sum for SWAN-I and SWAN-C. Note that high scores on the SWAN-I and SWAN-C are associated with worse attention and more ADHD-like behavior.

### Statistical methods

All analysis was performed using MATLAB and Statistics Toolbox Release 2012b, The MathWorks, Inc., Natick, Massachusetts, United States.

The variables included in analysis were as follows:

1. The average vector strength (VS).
2. The SWAN questionnaire, inattentive and combined scores (SWAN I and SWAN C).
3. The proportion of correct responses in the congruent and incongruent conditions of the flankers task (CONG and INC).
4. The mean reaction times in the EFT (RT CONG and RT INC).
5. The variability of reaction times in the EFT (RTV CONG and RTV INC).
6. The reaction time difference between the CONG and INC conditions in the EFT (RTD).

In order to evaluate the significance of the *VS* values, we used Rayleigh statistics, as provided by the circular statistics toolbox in MATLAB (Berens, 2009).

The distribution of VS scores was analyzed and found to be highly skewed (−2.9) with long tails revealed by the large kurtosis (13.5). Kurtosis is defined here as the fourth standardized moment of the distribution. Because of the non-normality of the distribution we decided to use the non-parametric Kruskall-Wallis analysis of variance to test differences across groups.

A Kruskall-Wallis test revealed a significant difference in *VS* across grades [χ^2^_(4, 97)_ = 21.25, *p* < 0.001]; therefore, we used grade as a covariate. Since VS scores do not vary linearly with grade, we treated grade as a nominal rather than continuous variable.

A Kruskall-Wallis test for differences across the two playing groups within the same grade revealed group differences only for grade 3 [χ^2^_(1, 14)_ = 7.46, *p* < 0.01]; therefore, we used playing group as a covariate for grade 3. Categorical and continuous data was combined to evaluate the effect of gender as a possible covariate. A value of 0 was assigned to female participants and a value of 1 to male participants, and the partial correlation between gender and the various scores was calculated (using covariates). Female participants were better synchronizers than male participants, reflected by the fact that gender correlated with VS scores [*r*_(100)_ = −0.22, *p* < 0.04]. Females were also more attentive [SWAN I *r*_(100)_ = 0.33, *p* < 0.01], less hyperactive [SWAN H *r*_(100)_ = 0.31, *p* < 0.01], more accurate on the incongruent flankers [INC *r*_(100)_ = −0.27, *p* < 0.02], and had lower reaction time in the congruent condition [RT CONG *r*_(100)_ = 0.26, *p* < 0.02]. Therefore, gender was used as a covariate in all further analyses.

Using these covariates, we then calculated the partial correlation between VS scores and each of the other variables (SWAN C, SWAN I, CONG, INC, RT CONG, RT INC, RTV CONG, RTV INC, RTD) and analyzed the distribution of the residuals. We found all distributions to be highly skewed (all skewness values were larger in magnitude than −2.2) and to have long tails (all kurtosis were larger than 10). Spearman's rank correlation was used for further analysis since it is more robust. Spearman's method ranks the data. Therefore, the variable distributions become symmetric and the influence of outliers is reduced. Spearman's correlation is generally considered to quantify the degree in which two variables are monotonically related. In the specific case of this study, it quantifies the degree in which better synchronizers tend to be better attenders (SWAN I), or perform better in psychometric task variables proposed to measure aspects of attention (EFT).

In order to evaluate the effect of this transformation, the distribution of residuals was analyzed under Spearman's method: all skewness values were below 0.26 and all kurtosis lay between 2.7 and 3.

The significance of the relationship between the different variables and gender remained in Spearman correlations, so gender continued to be used as a co-variate in our further analyses.

In summary, we ran Spearman's partial correlations using grade, gender, and group (on 3rd grade) as covariates.

## Results

**Figure 2:**
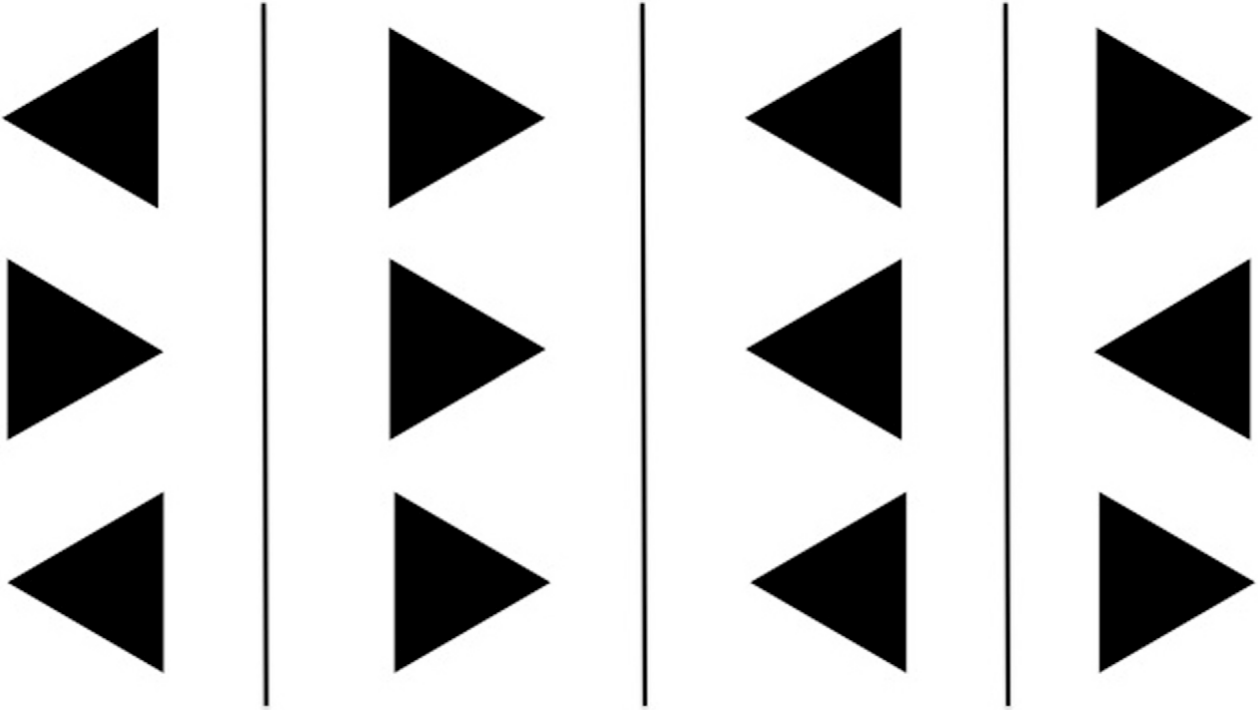
**The four sets of stimuli used in the Eriksen Flanker Task.** Left to right: right incongruent, right congruent, left congruent, and left incongruent. The flanking arrows appear 50 milliseconds before the center one.

**Figure 3:**
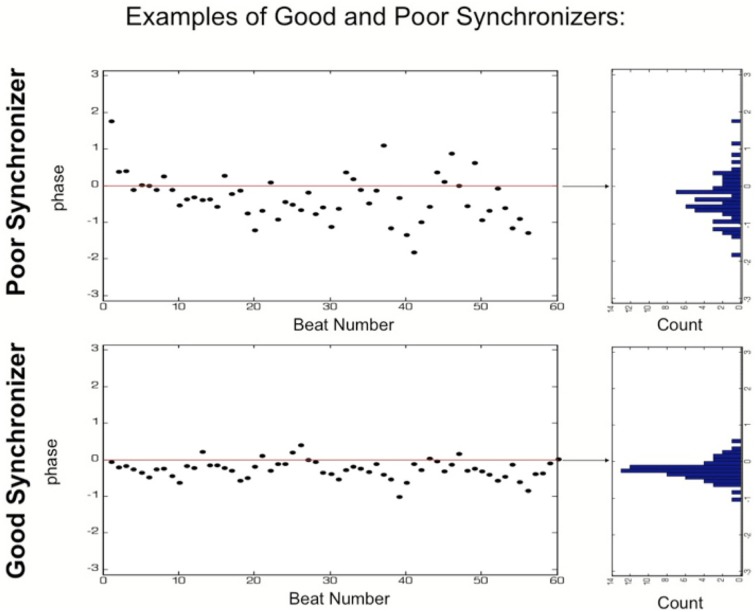
**Examples of good and poor synchronizers.** Mallet strikes, or onsets, corresponding to one synchronization episode of ~1 min. Each dot corresponds to one onset. If a dot is above “0” on the y axis, the corresponding mallet strike took place after that of the leader, if a dot is below “0,” the corresponding mallet strike took place before it. The right panel displays the spread of the phases. The good synchronizer **(bottom)** had very little spread relative to the poor synchronizer **(top)**. This spread was quantified using vector strength analysis (VS).

All participants in all episodes were significantly synchronized, as assessed by the Rayleigh statistics (*p* < 0.05). Figures 2, 3 presents an example of a strong synchronizer and a weak synchronizer. Table 1 shows summary statistics of the different variables for each grade. Table 2 shows the Spearman correlations between all variables. This is particularly compelling as some of the variables considered are highly correlated with each other, and 6 out of 9 of them are significantly correlated with *VS*.

**Table 2:** **Correlation matrix**.

	**SWAN I**	**SWAN C**	**CONG**	**INC**	**RT CONG**	**RT INC**	**RTV CONG**	**RTV INC**	**RTD**
VS	−0.41^***^	−0.42^***^	0.32^**^	0.15	−0.12	−0.057	−0.43^***^	−0.34^**^	0.22^*^
SWAN I		0.95^***^	−0.21	−0.13	0.084	0.0057	0.23^*^	0.18	−0.15
SWAN C			−0.19	−0.075	0.12	0.0085	0.27^*^	0.2	−0.22
CONG				0.81^***^	0.27^*^	0.34^**^	−0.41^***^	−0.62^***^	0.32^**^
INC					0.63^***^	0.6^***^	−0.057	−0.4^***^	0.13
RT CONG						0.94^***^	0.55^***^	0.23^*^	0.069
RT INC							0.47^***^	0.2	0.37^***^
RTV CONG								0.7^***^	−0.11
RTV INC									−0.075

It should be noted that bigger scores in the SWAN I and SWAN H scales are associated with poorer attention and higher hyperactivity respectively; both these variables are negatively correlated with the ability to synchronize. Higher variability in reaction time has also been associated with poorer attention. Individual correlations:

* p < 0.05,

** p < 0.01,

*** p < 0.001.

As shown in Table 2, a significant correlation emerged from the ability to synchronize as assessed by *VS* and most of the other variables analyzed. *VS* was correlated with both SWAN categories, the percentage of correct responses in the congruent condition of the EFT, the RTV in both the congruent and incongruent conditions, and the reaction time difference between congruent and incongruent conditions.

The effects we observed were not driven by data from participants at the extreme end of the continuum of the SWAN-C rating, presenting the strongest ADHD-like behaviors. When data from the 20% of participants who scored most poorly on the SWAN-C scale is removed from the analysis, a similar pattern of correlations can be observed. These correlations are generally smaller (Table 3), but this can expected since the data is truncated (Edwards, 1984).

**Table 3:** **Correlation matrix in which data from participants with the poorest attention scores has been excluded from analysis**.

	**SWAN I**	**SWAN C**	**CONG**	**INC**	**RT CONG**	**RT INC**	**RTV CONG**	**RTV INC**	**RTD**
VS	−0.35^**^	−0.38^***^	0.27^*^	0.14	−0.078	−0.031	−0.34^**^	−0.28^*^	0.16
SWAN I		0.92^***^	−0.2	−0.17	−0.04	−0.089	0.099	0.1	−0.079
SWAN C			−0.18	−0.094	0.028	−0.079	0.16	0.14	−0.19
CONG				0.81^***^	0.3^*^	0.36^**^	−0.37^**^	−0.62^***^	0.26^*^
INC					0.63^***^	0.59^***^	−0.042	−0.44^***^	0.031
RT CONG						0.92^***^	0.52^***^	0.15	−0.051
RT INC							0.46^***^	0.14	0.29^*^
RTV CONG								0.66^***^	−0.13
RTV INC									−0.11

The procedure was identical to that of Table 2 except that data from the 20% of children with the highest SWAN-C scores was excluded. Although the correlations are smaller, the same pattern as in Table 2 can be observed. This indicates that the statistics are not dominated by the children at the end of the spectrum but can be seen throughout.

## Discussion

Results from our investigation of 102 children aged 7–12 indicate that better synchronizers are also more attentive (SWAN-I), show less ADHD-like behaviors (SWAN-C), and are more accurate with lower RTV on the EFT.

Consistent with these findings is the hypothesis by Rolf that early development of attention in children relies on synchronous social interaction that emerges from effective audio-visual integration (Rolf et al., 2009). Some theoretical work considers the ability of joint action as essential to effective social timing (Pacherie and Dokic, 2006). While such work primarily relies on an analysis of the role of the mirror neuron system in joint action, Schmidt et al. (2011) make the point in a recent article that “Even if perception and action coding occurs in mirror systems as argued by cognitive theorists and such a representational system is the mechanism that allows us to understand another's actions, we still need to understand how joint actions are coordinated in time.” Our examination of the ability at rhythmic synchrony in a group context allows for experimental recording of precisely-timed conjoint action, while maintaining some ecological validity with respect to the classroom. Our finding of a significant correlation between rhythmic synchrony and attention behavior, as rated by teachers, provides an initial step toward establishing such an area of study.

The observed relationship between ability to synchronize and attention behavior may be explained in part by individual variability in the ability to generate rhythmic expectation. The capacity to synchronize is dependent upon the ability to generate expectation based on perceived patterns of temporal dynamics or rhythm, known as rhythmic expectation. The generation of rhythmic expectation enhances the ability of an individual to perform sensory discriminations at specific points in time. This aspect of attention, known as “dynamic attention” (Large and Jones, 1999), may play a role both in rhythmic performance and attentional behavior. Poor ability to generate rhythmic expectation can affect ability to modulate attention dynamically in accordance with temporal patterns created through joint action. This, in turn, could affect both ability to synchronize rhythmically and attention in interpersonal interaction.

In line with our findings showing a significant relationship between RTV and behavioral ratings of attention and also attention combined with hyperactivity-impulsivity (corresponding to attentional and combined diagnostic subtypes of ADHD, respectively), many studies have found increased RTV in those with ADHD on standard reaction time tasks [reviewed by Klein et al. (2006)]. Stimuli for such tasks are very frequently presented at a fixed interstimulus interval or ISI (e.g., continuous performance tests). Performance on such tasks, therefore, is partly dependent on the ability to synchronize because they require the generation of rhythmic expectations that enhance sensory discrimination at specific points in time, or when stimuli are presented (Large and Jones, 1999). The extensive literature on increased RTV in ADHD may then be attributable in part to impairment in the ability to synchronize.

Although the focus of this study is the relationship between rhythmic synchrony and *attentional* behavior, as measured by the inattentive components of the SWAN rating scale, we also found a significant correlation between rhythmic synchrony and *ADHD-like* behaviors as assessed by the combined (SWAN C) rating scale.

Much of the extant literature that examines the role of timing in relation to attention behavior focuses on differences between typically developing subjects and those with ADHD (Toplak et al., 2006). Such a focus tends to frame attention as a binary trait—something that a child either does or does not have. However, attentional behavior exists on a continuum, with the clinical population with ADHD occupying the extreme of the trait. We found that the significance of the correlation between the SWAN rating and VS holds across the full spectrum of attention behavior and rhythmic synchrony, even when the extremes of this continuum are eliminated from the analysis. This is important for our interpretation because it demonstrates that a relation between some aspects of temporal processing (and integration) and attention behavior is relevant to the entire population.

There are perhaps many subjacent deficits that can lead a child to exhibit behavior that teachers may perceive as associated with poor attention. It is important to understand what these subjacent deficits might be and identify behavioral biomarkers that can be associated with them. Based on our finding that the ability to synchronize in a group setting is correlated with teachers' perception of the attentional characteristics of a child, we pose that this ability can be one such biomarker. Further, this ability can be measured as it evolves in the context of a regular music class.

Synchronizing with others in a group music class context, aside from temporal processing and integration, also involves selective listening. Participants must be able to identify and focus on the target beat played by the leader. We attempted to minimize this by providing the teacher with an instrument that was significantly louder than, and had a very different timber than, the participants' instruments. In the future, it would be of interest to conduct a similar study that compares participants' synchrony in group and individual conditions and also includes a cognitive task for selective listening.

It could be argued that the observed correlation between rhythmic synchrony and SWAN ratings exists merely because, as with any cognitive task, those with poorer attention are less likely to be engaged. VS analysis (see Materials and methods) minimizes this effect because if a subject disengages completely (i.e., stops playing the instrument) the *VS* score remains unaffected, thus participants' synchrony was measured only when they were sufficiently engaged to be playing their instruments with the group. It should be stressed that we found that on all episodes the participants were performing the task, as shown by the fact that they were significantly synchronized.

Our study focused on attention in the classroom setting. Because our experimental design did not include measures of ability or achievement it is not known how the results obtained carry into those domains, although such comparisons will become important to our future work.

While the present study is one of numerous studies that have found a relationship between musical ability and other aspects of behavior (Forgeard et al., 2008; Kraus and Chandrasekaran, 2010), this study is unique in that it focuses on a specific—and quantifiable—component of musical performance: rhythmic synchrony. By measuring rhythmic synchrony directly, it is possible to find a continuum of individual differences in performance ability and correlate this continuum against other individual characteristics. Further, because music integrates multiple nested timescales, it is possible to extend the methodology we have developed to record participants individually in a group setting and measure their rhythmic synchrony to compare such things as sequence learning and synchrony, exploring multiple levels of synchrony across different timescales.

Although the idea that the practice of music positively influences general cognitive development appears entrenched in popular culture, there are few studies that have shown a causal relationship (Rauscher et al., 1997; Rauscher and Zupan, 2000; Schellenberg, 2004; Moreno et al., 2009). None of these intervention studies make clear what specific aspects of music practice might contribute to the observed changes. Parsing components of music practice (such as synchrony, sequencing, and pitch matching) in such a way that their role in possible cognitive changes can be measured will be an important step both for scientific study of music and for the development of specific music programs. This study is correlational and cannot test whether a causal relationship exists between music and attention. However, causation cannot exist without correlation and thus establishing the existence of a correlation with a specific and measurable component of music practice is a necessary step toward investigating causation before entering into complex and costly intervention studies.

The key finding of this study is the significant correlation between rhythmic synchrony and attention behavior. Many studies have investigated correlations between the practice of music and various elements of cognition (Kraus and Chandrasekaran, 2010). The present study, however, rather than investigating music practice in general, quantifies a specific component of music: rhythmic synchrony. We hope that this study and its methodology will point toward further quantifiable investigation of relationships between music and cognition.

### Conflict of interest statement

The authors declare that the research was conducted in the absence of any commercial or financial relationships that could be construed as a potential conflict of interest.

## References

[B1] Albrecht B. Brandeis D. Uebel H. Heinrich H. Mueller U. C. Hasselhorn M. (2008). Action monitoring in boys with attention-deficit/hyperactivity disorder, their nonaffected siblings, and normal control subjects: Evidence for an endophenotype. Biol. Psychiatry 64, 615–625 10.1016/j.biopsych.2007.12.016 18339358 PMC2580803

[B2] Arnett A. B. Pennington B. F. Willcutt E. Dmitrieva J. Byrne B. Samuelsson S. (2012). A cross-lagged model of the development of ADHD inattention symptoms and rapid naming speed. J. Abnorm. Child Psychol. 40, 1313–1326 10.1007/s10802-012-9644-5 22581405 PMC3546520

[B3] Barkley R. A. Murphy K. R. (2006). Attention-Deficit Hyperactivity Disorder: A Clinical Workbook, Vol. 2. New York, NY: Guilford Press

[B4] Berens P. (2009). CircStat: a MATLAB toolbox for circular statistics. J. Stat. Softw. 31, 1–21

[B5] Bernieri F. J. Rosenthal R. (1991). “Interpersonal coordination: Behavior matching and interactional synchrony,” in Fundamentals of nonverbal behavior. Studies in emotion and social interaction, eds Feldman R. S. Rime B. (New York, NY: Cambridge University Press), 401–432

[B6] Castellanos F. X. Tannock R. (2002). Neuroscience of attention-deficit/hyperactivity disorder: the search for endophenotypes. Nat. Rev. Neurosci. 3, 617–628 12154363 10.1038/nrn896

[B7] de Barbaro K. Chiba A. Deák G. O. (2000). MicroĆÄêanalysis of infant looking in a naturalistic social setting: insights from biologically based models of attention. Dev. Sci. 14, 1150–1160 10.1111/j.1467-7687.2011.01066.x 21884330

[B8] Deák G. O. Flom R. A. Pick A. D. (2000). Effects of gesture and target on 12-and 18-month-olds' joint visual attention to objects in front of or behind them. Dev. Psychol. 36, 511 10.1037/0012-1649.36.4.511 10902702

[B9] DSM-IV, APA. (1994). Diagnostic and Statistical Manual of Mental Disorders: DSM-IV. New York, NY: Amer Psychiatric Pub Inc

[B10] Edwards A. (1984). An Introduction to Linear Regression and Correlation. New York, NY: WH Freeman and Company

[B11] Eriksen C. W. (1995). The flankers task and response competition: a useful tool for investigating a variety of cognitive problems. Vis. Cogn. 2, 101–118 10.1080/13506289508401726

[B12] Forgeard M. Winner E. Norton A. Schlaug G. (2008). Practicing a musical instrument in childhood is associated with enhanced verbal ability and nonverbal reasoning. PLoS ONE 3:e3566 10.1371/journal.pone.0003566 18958177 PMC2570220

[B13] Gilden D. L. Marusich L. R. (2009). Contraction of time in attention-deficit hyperactivity disorder. Neuropsychology 23, 265 10.1037/a0014553 19254099

[B14] Goldberg J. M. Brown P. B. (1968). Functional organization of the dog superior olivary complex: an anatomical and electrophysiological study. J. Neurophysiol. 31, 639–656 5709877 10.1152/jn.1968.31.4.639

[B15] Jacoby N. Repp B. H. (2012). A general linear framework for the comparison and evaluation of models of sensorimotor synchronization. Biol. Cybern. 106, 135–154 10.1007/s00422-012-0482-x 22526355

[B16] Klein C. Wendling K. Huettner P. Ruder H. Peper M. (2006). Intra-subject variability in attention-deficit hyperactivity disorder. Biol. Psychiatry 60, 1088–1097 10.1016/j.biopsych.2006.04.003 16806097

[B17] Kraus N. Chandrasekaran B. (2010). Music training for the development of auditory skills. Nat. Rev. Neurosci. 11, 599–605 10.1038/nrn2882 20648064

[B18] Large E. W. Jones M. R. (1999). The dynamics of attending: how people track time-varying events. Psychol. Rev. 106, 119 10.1037/0033-295X.106.1.119

[B19] Moreno S. Marques C. Santos A. Santos M. Besson M. (2009). Musical training influences linguistic abilities in 8-year-old children: more evidence for brain plasticity. Cereb. Cortex 19, 712–723 10.1093/cercor/bhn120 18832336

[B20] Pacherie E. Dokic J. R. M. (2006). From mirror neurons to joint actions. Cogn. Syst. Res. 7, 101–112 10.1016/j.cogsys.2005.11.012

[B21] Rauscher F. H. Shaw G. L. Levine L. J. Wright E. L. Dennis W. R. Newcomb R. L. (1997). Music training causes long-term enhancement of preschool children's spatial-temporal reasoning. Neurol. Res. 19, 2–8 9090630 10.1080/01616412.1997.11740765

[B22] Rauscher F. H. Zupan M. A. (2000). Classroom keyboard instruction improves kindergarten childrenĆÄôs spatial-temporal performance: a field experiment. Early Child. Res. Q 15, 215–228 10.1016/S0885-200600050-8

[B23] Repp B. H. (2005). Sensorimotor synchronization: a review of the tapping literature. Psychon. Bull. Rev. 12, 969–992 10.3758/BF03206433 16615317

[B24] Rolf M. Hanheide M. Rohlfing K. J. (2009). Attention via synchrony: making use of multimodal cues in social learning. IEEE Trans. Auton. Ment. Dev. 1, 55–67 10.1109/TAMD.2009.2021091

[B25] Schellenberg E. G. (2004). Music lessons enhance IQ. Psychol. Sci. 15, 511–514 10.1111/j.0956-7976.2004.00711.x 15270994

[B26] Schmidt R. Fitzpatrick P. Caron R. Mergeche J. (2011). Understanding social motor coordination. Hum. Mov. Sci. 30, 834–845 10.1016/j.humov.2010.05.014 20817320

[B27] Swanson J. Schuck S. Mann M. Carlson C. Hartman K. Sergeant J. (2006). Categorical and Dimensional Definitions and Evaluations of Symptoms of ADHD: The SNAP and the SWAN Rating Scales. Available online at: http://www.adhd.net/SNAP_SWAN.pdf PMC4618695 26504617

[B28] Toplak M. E. Dockstader C. Tannock R. (2006). Temporal information processing in ADHD: findings to date and new methods. J. Neurosci. Methods 151, 15–29 10.1016/j.jneumeth.2005.09.018 16378641

